# Development of chitosan-based biodegradable films enriched with thyme essential oil and additives for potential applications in packaging of fresh collard greens

**DOI:** 10.1038/s41598-022-20751-1

**Published:** 2022-10-08

**Authors:** Aiman Zehra, Sajad Mohd Wani, Nusrat Jan, Tashooq Ahmad Bhat, Sajad A. Rather, A. R. Malik, Syed Zameer Hussain

**Affiliations:** 1 Division of Food Science and Technology, Sher-e-Kashmir University of Agricultural Sciences and Technology, Kashmir, 190025 India; 2 Department of Food Science and Technology, University of Kashmir, Hazratbal, Srinagar, 190006 India; 3 Division of Fruit Science, Sher-e-Kashmir University of Agricultural Sciences and Technology, Kashmir, 190025 India

**Keywords:** Microbiology, Plant sciences

## Abstract

In the present study, chitosan (CH) based biodegradable films were developed enriched with thyme essential oil (TEO) incorporated with different additives including zinc oxide (ZnO), polyethylene glycol (PEG), nano clay (NC), and calcium chloride (CaCl_2_) and characterize the postharvest quality of ‘collard greens’ during refrigerated storage. The results indicated that the incorporation of ZnO/PEG/NC/CaCl_2_ in CH-based films significantly decreased water vapor transmission rate, increased tensile strength, and were water soluble and biodegradable in nature. Moreover, CH-TEO based films incorporated with ZnO/PEG/NC/CaCl_2_ were significantly effective in reducing physiological weight loss, retained total soluble solids, titratable acidity, and preserved chlorophyll contents as well as showed lesser a* values, suppressed microbial growth, and preserving appearance/sensory quality of collard greens for 24 days than LDPE and other biodegradable films. Our results suggest that CH-based films enriched with TEO and additives such as ZnO/CaCl_2_/NC/PEG are an ecological, environmental friendly, and effective alternative approach to retain shelf life of collard greens during refrigerated storage.

## Introduction

Synthetic polymeric packaging materials derived from petroleum have long been used in the food sector for ensuring the quality and safety of a wide range of foods. The benefits of such conventional substances are clear due to their ease of production, low cost, and excellent barrier properties. However, the extensive use and dumping of these non-degradable substances inevitably exacerbate the rising crisis of pollution in the environment. Under this circumstance, the development of natural eco-friendly packaging materials has increased tremendously in recent years. These novel films are non-toxic, biodegradable, sustainable, and biocompatible^1^. Apart from being non-toxic and biocompatible, these naturally derived biopolymer-based films can transport antioxidants and therefore, do not create any inherent contamination to food including the leaching of additives like phthalates^2^. Thus, these substrates can be used as a viable substitute for conventional petroleum-based plastics, owing to their similar functional features for food packaging^3^. Nowadays, biopolymers derived from proteins, lipids, and polysaccharides have been effectively exploited to constitute a series of new environmentally friendly packaging substances. Chitosan (CH) has been extensively used in food packaging applications among the plentiful natural macromolecules including cellulose and starch pertaining to the polysaccharide class owing to its easy film-forming capacity, biodegradability, better oxygen, and water vapor barrier ability, and good mechanical strength^4,5^. However, the low antioxidant and antibacterial potentials of CH film, which are key criteria for an active food packaging film, limit its potential^6^, thus, additional molecules have been incorporated into CH film to generate novel substances with appropriate applicability^7^.

Essential oils derived from plants can be incorporated into biopolymer films and can furnish packaging systems with antioxidant or antimicrobial properties, which is beneficial to prolong the shelf life of food commodities. Thyme (*Thymus vulgaris*) essential oil is by far the most examined and used essential oil due to its antibacterial, anti-inflammatory, and antifungal characteristics^8^. Various thyme chemotypes have been identified based on compositions of essential oil, which include thymol (23–60%), *p*-cymene (8–44%), γ-terpinene (18–50%), linalool (3–4%) and carvacrol (2–8%)^9^, however, thymol has the strongest antimicrobial action due to their phenolic composition^10^. Unfortunately, incorporating plant essential oils or their active constituents into biopolymer matrixes substantially results in a significant reduction in the mechanical strength of the obtained bio-composite films^11,12^. This implies that wrapping materials and plasticized films containing plant essential oil components should undergo additional reinforcement treatment in order to enhance their mechanical properties for food packaging.

Different additives can be involved to enhance the mechanical and structural properties of developed biocomposite films. It has been evidenced that additives such as calcium chloride (CaCl_2_), nano clay (NC), polyethylene glycol (PEG), and zinc oxide (ZnO) have been shown to help in enhancing the antibacterial and gas barrier qualities of active food packaging films, as well as control resistance of packaging for humidity and temperature^13,14^. According to Hari et al.^15^, CaCl_2_ and ZnO have shown high antibacterial activity as well as a positive influence on the barrier and mechanical characteristics of biocomposite films. Furthermore, ZnO, CaCl_2_, NC, and PEG have outstanding antioxidant and photo-catalytic characteristics, in addition to being non-toxic and more stable in nature and having a high surface-to-volume ratio and extraordinary surface reactivity^16,17^. ZnO, CaCl_2_, NC, and PEG have been widely used as safe ingredients in the drug, food, and cosmetic items due to their aforementioned functional capabilities, and their food safety (GRAS—Generally Recognized as Safe) has been properly authorized by the Food and Drug Administration (FDA) of the United States^18,19^. To the best of our knowledge, the inclusion of different additives viz., ZnO, CaCl_2_, NC, and PEG in CH-TEO-based films has not been investigated till date for the purpose outlined above. Therefore objective of this study was development of CH-based films with incorporation of TEO as n antimicrobial agent and ZnO/PEG/NC/CaCl_2_ as additives and to investigate its effect on the postharvest quality of collard greens.

## Materials and methods

### Chemicals and reagents

Chitosan (low molecular weight, DD ≥ 98%) was purchased from Hi-Media India. TEO was procured from the local market of Srinagar, Kashmir. The raw material was procured from the Sher-e-Kashmir University of Agricultural Sciences and Technology-Kashmir, and all the methods used in this work are in compliance with the institutional guidelines. Glycerol (56-81-5), acetic acid (64-19-7), tween 80 (9005-65-6), PEG (25322-68-3), CaCl_2_ (10043-52-4), ZnO (1314-13-2), NC (1302-78-9), and potassium hydroxide (KOH) (1310–58-3) were purchased from Sigma-Aldrich. All other chemicals and reagents used in this research were of analytical grade and purchased from Hi-Media Pvt. Limited India.

### Preparation of films

The CH films were prepared according to the protocol illustrated by Wongpanit et al.^20^ with slight modifications. One gram (1 g) of CH was dissolved in 100 mL of distilled water followed by the addition of 1% of acetic acid. Then 1% (v/v) of glycerol was added as a plasticizer and 2–3 drops of tween 80 were added as an emulsifier to the mixture. The reaction mixture was heated until completely dissolved in order to get a clear and homogeneous solution. Film-forming solutions were sonicated for 30 min before use to remove bubbles. TEO (1 g/100 mL) as an antimicrobial agent was also incorporated in the CH films. The additives CaCl_2_, PEG, NC, and ZnO were added in the proportions of 0.12 g/100 mL, 2 g/100 mL, 0.05 g/100 mL and 0.05 g/100 mL, respectively in different combinations. Then the known volume of the film solution was cast on the polypropylene petri dishes and was dried at 40 ± 5 °C for 42 h. The resulting dried films were then neutralized in a 20 mL solution of 0.1 N KOH for 15 min under slight shaking at room temperature, washed with distilled water, and left to dry for 1 h. A total combination of 16 different biodegradable films was formed (Table 1) and were conditioned in a humidity chamber at 50 ± 5% relative humidity and 23 ± 2 °C. LDPE was taken as a control film where no treatment was being added.

**Table 1:** Composition of different chitosan-based biodegradable films.

Packaging combinations	Treatments	Composition
LDPE (control)	(T0)	Nil
Chitosan (CH) + thyme essential oil (TEO)	(T1)	1 g/100 mL + 1 mL/100 mL
CH-TEO + calcium chloride (CaCl_2_)	(T2)	1 g/100 mL + 1 mL/100 mL + 0.2 g/100 mL
CH-TEO + polyethylene glycol (PEG)	(T3)	1 g/100 mL + 1 mL/100 mL + 2 g/100 mL
CH-TEO + nano-clay (NC)	(T4)	1 g/100 mL + 1 mL/100 mL + 0.05 g/100 mL
CH-TEO + zinc-oxide (ZnO)	(T5)	1 g/100 mL + 1 mL/100 mL + 0.05 g/100 mL
CH-TEO + PEG/CaCl_2_	(T6)	1 g/100 mL + 1 mL/100 mL + 2 g/100 mL + 0.2 g/100 mL
CH-TEO + NC/CaCl_2_	(T7)	1 g/100 mL + 1 mL/100 mL + 0.05 g/100 mL + 0.2 g/100 mL
CH-TEO + ZnO/CaCl_2_	(T8)	1 g/100 mL + 1 mL/100 mL + 0.05 g/100 mL + 0.2 g/100 mL
CH-TEO + NC/PEG	(T9)	1 g/100 mL + 1 mL/100 mL + 0.05 g/100 mL + 2 g/100 mL
CH-TEO + PEG/ZnO	(T10)	1 g/100 mL + 1 mL/100 mL + 2 g/100 mL + 0.05 g/100 mL
CH-TEO + NC/ZnO	(T11)	1 g/100 mL + 1 mL/100 mL + 0.05 g/100 mL + 0.05 g/100 mL
CH-TEO + CaCl_2_/PEG/NC	(T12)	1 g/100 mL + 1 mL/100 mL + 0.2 g/100 mL + 2 g/100 mL + 0.05 g/100 mL
CH-TEO + NC/ZnO/CaCl_2_	(T13)	1 g/100 mL + 1 mL/100 mL + 0.05 g/100 mL + 0.05 g/100 mL + 0.2 g/100 mL
CH-TEO + ZnO/PEG/NC	(T14)	1 g/100 mL + 1 mL/100 mL + 0.05 g/100 mL + 2 g/100 mL + 0.05 g/100 mL
CH-TEO + ZnO/CaCl_2_/PEG	(T15)	1 g/100 mL + 1 mL/100 mL + 0.05 g/100 mL + 0.2 g/100 mL + 2 g/100 mL
CH-TEO + NC/ZnO/PEG/CaCl_2_	(T16)	1 g/100 mL + 1 mL/100 mL + 0.05 g/100 mL + 0.05 g/100 mL + 2 g/100 mL + 0.2 g/100 mL

### Sample preparation

The leaves of collard greens were harvested manually from the experimental farm of SKUAST-K on 18-04-2021 and were transported on the same date to the laboratory of Food Science and Technology, SKUAST-K. The leaves were washed with distilled water in order to remove any soil particles and were placed on sanitized trays of polypropylene until the surface moisture dried completely. Then the known weight of leaves was packed in 16 different biodegradable films and LDPE film (Plate S). The collard green leafy vegetables were stored for 24 days under refrigerated conditions and were evaluated at 0, 4, 8, 12, 16, 20, and 24th days of storage.

## Characterization of different CH-based films

### Mechanical property

Tensile strength (MPa) was determined at 25 °C with a TAXT Plus Texture Analyzer (Stable Microsystems, UK) according to ASTM-D-882-91^21^. Three dumb bell shaped test specimens with dimensions of 8 × 0.5 cm were prepared from each of the film samples and conditioned at 50% RH and 25 °C for 48 h before the evaluation. The initial grip separation and cross-head speed were set to 30 mm and 5 mm/min, respectively.

### Biodegradation analysis

The biodegradation test of prepared films was investigated according to the protocol reported by Sarojni et al.^22^. Biodegradability was determined by measuring the weight loss of the films buried under the soil. For the biodegradability analysis, the films were cut into 5 × 2 cm pieces, weighed, tied at one corner with a thread, and buried at about 2–3 cm below the surface of the soil. The humidity of the soil was regulated by sprinkling tap water onto the soil once a day. After different intervals of time, the pre-weighed films were removed from the soil, washed with distilled water several times to clean off the soil particles, and dried at room temperature to constant weight. The weight loss was then calculated using the following equation;

$$Degradation\,\, \left(\%\right)=\frac{W1-W2}{W1}$$

where, W1 is the initial dry weight of the sample and W2 is the dry weight of the sample after degradation test in soil.

### Water vapor transmission rate

Water vapor transmission rate was measured using a modified ASTM 96-00 method^23^. The film was sealed on a modified test cell containing 15 mL of distilled water. The test cell was then kept in a desiccator containing pre-dehydrated silica gel. Silica gels were dried at 180 °C for 3 h for these measurements. The whole assembly was kept at 25 °C and weight loss of the test cell was measured after storage for 24 h. WVTR of the film was calculated using the following equation;

$$WVTR= \Delta t \times A$$

where ΔW is the weight loss of test cell, Δt is the time of storage, and A is the area of exposed film.

### Water solubility

Water solubility was determined according to the method reported by Rubilar et al.^24^. Solubility is defined as the content of dry matter solubilized after 24 h immersion in water. The initial dry matter content of each film was determined by drying to constant weight in an oven at 105 °C. Film disks (2 cm diameter) were cut, weighed (Mi), and immersed in 50 mL of water. After 24 h of immersion at 20 °C with agitation (60 rpm), the samples were taken out and dried to constant weight (Mf) in an oven at 105 °C, to determine the weight of dry matter that was not solubilized in water. The solubility of each film was then calculated using the following equation:

$$Water solubility\,\, \left(\%\right)= \frac{Mi-Mf}{Mi}\times 100$$

where, Mi is the initial mass and Mf is the final mass of the sample.

## Characterization of fresh collard greens packed in different CH-based films

### Physiological weight loss

The weight of whole package including collard greens on each day of analysis was measured by using a laboratory-scale digital weighing balance (SHIMADZU AUY120). Physiological weight losses (PWL) were determined by comparing weight of samples before and after the storage period. The results were represented as weight loss percentage with regard to the initial weight^25^.

$$\mathrm{PWL }(\mathrm{\%}) = \frac{{W}_{in}-{W}_{fin}}{{W}_{in}} \times 100$$

where *W*in is the weight of the first day and *W*fin the weight of final day.

### Total chlorophyll content

Total chlorophyll content of collard greens was determined by the method followed by Gu et al.^26^. Fresh sample of 0.5 g was macerated in pestle and mortar. Then 2 mL of ethanol was mixed with 4 mL of 99% acetone in the ratio of 1:2 v/v and added to sample. The solution was kept in a freezer and left to stand for 30 min and centrifuged at 2000 rpm for 10 min. Again 5 mL of ethanol/ acetone (1:2 v/v) was added and stirred for 1 min. Absorbance was taken using a spectrophotometer (UV-2450 Shimadzu, Japan) at wavelengths of 663 nm and 645 nm (detecting chlorophyll a and chlorophyll b, respectively). The ratio of ethanol/acetone (1:2 v/v) was taken as control. The photosynthetic pigments present in the sample were estimated by the following formulas:

$$ \begin{gathered} {\text{Chlorophyll a }}\left( {{\text{mg}}/{\text{g}}} \right)\, = \,\left( {{12}.{7 }*{\text{ A663}}} \right) - ({2}.{59 }*{\text{ A645}}) \hfill \\ {\text{Chlorophyll b }}\left( {{\text{mg}}/{\text{g}}} \right)\, = \,\left( {{22}.{9 }*{\text{ A645}}} \right) - ({4}.{7 }*{\text{ A663}}) \hfill \\ {\text{Total chlorophyll }}\left( {{\text{mg}}/{\text{g}}} \right)\, = \,\left( {{8}.{2 }*{\text{ A663}}} \right)\, + \,({2}0.{2 }*{\text{ A645}}) \hfill \\ \end{gathered} $$

where A663 and A645 are the absorbances measured at 663 nm and 645 nm, respectively.

The spectrophotometer was adjusted to zero using the ethanol/acetone mixture. The results were represented as mg chlorophyll/g FW.

### Total soluble solids and titratable acidity

Total soluble solids of collard greens were performed by refractometer (Atago RX-1000). The sample was prepared by macerating 1 g of the sample and diluted in 1 mL of distilled water. The results were represented in the degree Brix in accordance with the Association of Official Analytical Chemistry^27^.

Titratable acidity of collard greens was determined according to the method of Schvambach et al.^25^. One gram of sample was macerated with 75 mL of distilled water using phenolphthalein indicator and titrated with 0.1 M NaOH. The results were represented as percent of malic acid.

### Color evaluation

Hunter Lab colorimeter with micro-sensors (Model SN3001476, New York) was used to determine the color of the samples. Hunter values for L* (Lightness), a* (redness), and b* (yellowness) were used to express color readings. The calorimeter calibration was performed using black and white reference plates.

### Microbiological assessment

The yeast and mould count was determined using a standard serial dilution procedure with potato dextrose agar as growth medium. The sample (10 g) was homogenized in sterile 0.1% peptone water (90 mL) to make a suspension and was serially diluted up to 10^–4^. Then 1 mL of each dilution was inoculated in duplicate into sterile petri dishes using pour plate technique. The plates were incubated at 28 ± 2 °C for 2–7 days and colony counts were recorded. The results were represented as a logarithm of colony-forming units (log cfu/g)^28^. Similarly total plate count (TPC) was determined using a standard serial dilution procedure with plate count agar as growth medium. The data were represented as a logarithm of colony-forming units (log cfu/g)^29^.

### Sensory evaluation

The collard green leaves were evaluated for sensory attributes (overall acceptability). Acceptance tests were performed by a panel of 30 panellists between the ages of 20–45 to measure the level of liking or dislike using a 5-point hedonic rating scale.

### Statistical analysis

The experiments were repeated three times, results were expressed in the form of “mean ± standard deviation”. SPSS (24.0) software was used to analyze the significance of the data, and the significance level was p ≤ 0.05.

## Results and discussion

### Characterization of different CH-based films

#### Mechanical property

**Figure 1:**
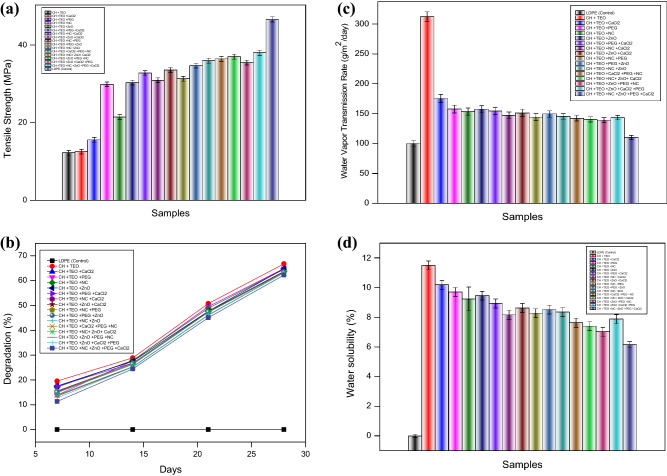
(**a**) Tensile strength of control and chitosan-based biodegradable films. (**b)** Biodegradability of control and chitosan-based films. (**c)** Water vapour transmission rate of different chitosan-based films. (**d)** Water solubility of different chitosan-based films.

The mechanical properties reflect the durability of the films and their ability to maintain the integrity of packaged food products during handling and storage^30^. The effect of incorporating TEO and different additives on TS of CH-based films is presented in Fig. 1a. The results indicated that the TS of CH-TEO-based film (T_1_) was low (12.24 MPa) as compared to control and other biodegradable films. The incorporation of TEO induced a decrease in the TS of CH films. It was proposed that the addition of a hydrophobic agent to the film composition induces the development of structural discontinuities, producing a film structure with less chain mobility, and consequently, with less flexibility and resistance to fracture^31^. Generally, oil addition to polysaccharide films decreases TS values, once lipids are not capable to form continuous and cohesive matrices^32^. Previously, Moradi et al.^33^ reported a reduction in CH film extensibility and TS after the incorporation of *Zataria multiflora* essential oil, which was attributed to a rise in pore sizes of the films, thereby supporting our results. On the other hand, TS of CH-TEO-based films significantly increased with the incorporation of NC, ZnO, PEG, and CaCl_2_. This effect might be due to the hydrogen bonding interactions between NC, ZnO, PEG, CaCl_2_-OH, and amine groups of CH. Highest TS was observed in the films incorporated with CH-TEO and ZnO/PEG/NC/CaCl_2_ (T_16_) which might be due to the formation of strong hydrogen bonds between CH and ZnO/PEG/NC/CaCl_2_ particles. It is interesting to note that on account of the intermolecular interactions and mechanical strength, the blended composite film (T_16_) was stronger than the CH-TEO film and other biodegradable films probably due to the presence of different additives. Similar results were claimed by Hiremani et al.^34^ and Sarojni et al.^22^.

#### Biodegradation analysis

The biodegradation of the films was determined by changes in the weight of the film. The biodegradation rates of the film, measured by weight loss at different time intervals, are shown in Fig. 1b. The data depicted that the LDPE film showed least biodegradability compared to CH-TEO film and other films incorporated with ZnO, PEG, NC, and CaCl_2_. The LDPE showed least biodegradability due to its chemical units in monomers that are linked together by forming extremely strong carbon–carbon bonds with each other that result in polymers with long chains of monomers called polyethylene^35^. The biodegradability of the materials is mainly dependent on humidity and chemical structure. After 28 days, CH-TEO film showed higher biodegradability as compared to other composite films which may be due to its hydrophilic nature. The moisture of the soil could easily penetrate the polymer network, thereby, weakening the polymer chains and making the susceptible to hydrolysis at the glycosidic linkages by soil microorganisms^22^. The extent of degradation of the composite films incorporated with ZnO/PEG/NC/CaCl_2_ was least among all the fabricated films throughout 28 days suggesting that the chemical bonds in the polymer were not easily amenable for biodegradation. Moreover, the observed degradation in T_16_ film (CH-TEO + ZnO/PEG/NC/CaCl_2_) might have been initiated by the presence of the hydrolysable aliphatic polyester bonds in the essential oil backbone^36^. These observations suggested that the biodegradability of all the composite films was markedly influenced by the presence of additives (ZnO, PEG, NC, and CaCl_2_) in them. Sarojni et al.^22^ claimed that mahua oil-based polyurethane/chitosan/nano ZnO composite films were biodegraded in soil up to 50% in 28 days. Similar results were reported by Hiremani et al.^34^ who claimed that the incorporation of piper betel leaf extracts enhanced the biodegradability of polyvinyl alcohol-oxidized maize starch blend films.

#### Water vapor transmission rate

The water vapor transmission rate (WVTR) values of the control film and composite films are presented in Fig. 1c. The control film (T_0_) presented a WVTR value of 100 g/m^2^/day while CH-TEO-based film presented a WVTR value of 312.32 g/m^2^/day. The results showed that the incorporation of TEO had a significant effect on the WVTR of CH films. This result may be explained by the hydrophobic nature of thymol, which affects the hydrophilic/hydrophobic balance of the film^24^. However, WVTR was decreased with the incorporation of different additives, and the lowest value for WVTR (110 g/m^2^/day) was examined for the treatment T_16_ (ZnO/PEG/NC/CaCl_2_) probably due to the presence of different additives. The combination of ZnO/PEG/NC/CaCl_2_ remarkably promotes a synergistic effect on the mechanical and barrier properties of the films. The presence of NC in the composite films formed tortuous pathways for the diffusion of water molecules, thereby increasing their average path lengths for passing through the film matrix. A similar report was claimed by Lee et al.^37^. The presence of ZnO porosity in the polymer surface leads to low WVTR^38^. The addition of PEG, despite also being a hydrophilic polymer decreased WVTR. The introduction of PEG in the film possibly facilitated the mobility of the polymer chains which would improve the dispersion of the nanoclays within the polymer films, increasing the TS. The higher mobility of the polymer chains, on the other hand, improves the ductility and barrier properties of the films^39^. The CaCl_2_ forms a strong interaction with CH polymer films by hydrogen bonding and this interaction was enhanced with the content of CaCl_2_ with other additives^40^. Thus the inclusion of NC/ZnO/PEG/CaCl_2_ in CH-TEO films significantly improved WVTR.

#### Water solubility

Water solubility (WS) is a measure of the resistance of a film sample to water. It is an important parameter that determines the biodegradability of the film when used as a packaging material^41^. Higher or complete solubility of films can be helpful in degradation, while lower solubility of films is applicable for storage^34^. Figure 1d shows the solubility percentage of all the prepared films. The results showed that the highest WS was observed for CH-TEO-based film (11.5%). However, WS was decreased with the incorporation of different additives. This might be due to the occurrence of polymer interaction restricting the mobility of the polymer chain, thus decreasing the hydrophilicity of the film leading to reduce water affinity^42^. Meanwhile, the T_16_ film shows a lower water solubility rate (6.15%) in contrast with CH-TEO film and other biodegradable films which can be attributed to the lesser availability of free hydroxyl groups in the blend matrix to interact with water molecules. Moreover, the incorporation of TEO resulted in a lower WS due to the hydrophobic nature of the EO which further reduced the WS of the films.

### Characterization of fresh collard greens packed in different CH-based films

#### Physiological weight loss

**Figure 2:**
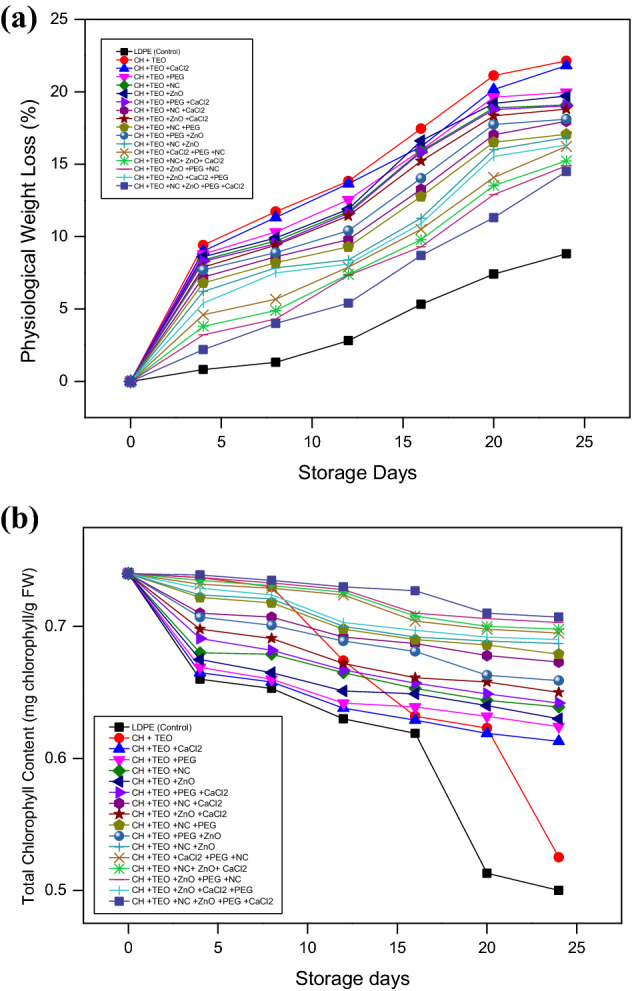
(**a**) Effect of different chitosan-based films and storage days on physiological weight loss (%) of collard greens. (**b)** Effect of different chitosan-based films and storage days on total chlorophyll (mg chlorophyll/g FW) content of collard greens.

The physiological weight loss (PWL) of collard greens under different packaging treatments during 24 days of storage is presented in Fig. 2a. The data revealed that PWL of collard greens increased significantly (p ≤ 0.05) among all the packing combinations during the entire storage period. At 0 day of storage, negligible PWL was observed in all collard greens with different packaging film treatments. However, at the end of the storage period, lower PWL was noticed in collard greens packaged in control (LDPE) film while higher PWL was noticed in collard greens (T_1_) packaged in CH films. The water WVTR of LDPE film is very less compared to that of biodegradable films due to which an increase in the PWL was observed among the collard greens packaged in biodegradable films. This WVTR factor might be related to the non-polar characteristic of LDPE, which makes it a hydrophobic polymer, in contrast to the polar nature of CH film^43^. Furthermore, lowest PWL was observed in the collard greens packaged in films incorporated with NC followed by ZnO, PEG, and CaCl_2._ This effect might be due to the enhanced non-polar characteristic of CH films incorporated with NC thus, decreasing the PWL in collard greens and increasing the water vapor barrier characteristics of films^43^. It has also been reported that with an increasing amount of incorporated clay into the CH films, the pathway for water molecules becomes more complex/longer. The longer the pathway created for the gas as well as the water vapor molecules to travel, the lesser will be the PWL^44^. The improvement in the barrier properties via nano clay incorporation in the CH-based films has been reported by earlier researchers^45,46^. On the other hand, T_16_ film showed much lower PWL in collard greens than other treatments as the CH films in this treatment were incorporated with ZnO/PEG/NC/CaCl_2_ which had a synergetic effect on the film physicochemical characteristics. CH-TEO-based films incorporated with ZnO/PEG/NC/CaCl_2_ limit water vaporization by creating a layer on the surface and reducing metabolic processes and respiration^47^, which was the principal cause for less reduction in PWL across the treatments. Moreover, essential oil action on the fruit surface can result in lower ethylene generation and respiration rates^48^ which reduce PWL during storage. A similar increase was also observed by Brandelero et al.^49^ when the lettuce was stored in polyvinyl chloride (PVC) and starch/PVOH/alginate films during storage, who recorded only 2% mass loss in PVC while 14–16% loss in starch/PVOH/alginate films during eight days of storage.

#### Total chlorophyll content

Chlorophyll is the primary source of green color in leafy vegetables and is the most important criteria for consumer’s perception. Figure 2b presented the total chlorophyll content (TCC) of collard greens under different packaging treatments during 24 days of storage. The data revealed that TCC of collard greens declined significantly (p ≤ 0.05) during the entire storage period irrespective of packaging film. At 0 days of storage, negligible loss of TCC was observed in collard greens among different packaging films. However, at the end of the storage period, T_16_ recorded the lowest loss of TCC while, T_1_ showed the highest loss of TCC (p ≤ 0.05). The main mechanisms of chlorophyll degradation are pH variations primarily because of spillage of organic acids from the vacuole, and activity of chlorophyllase enzyme^50^. Results of this study showed that the collard greens stored in LDPE showed a greater decrease in chlorophyll content after 8th day of storage in refrigerator. This observation could be explained by increased ethylene levels, which increase chlorophyllase activity, resulting in chlorophyll degradation^51^. Manolopoulou and Varzakas^52^ reported similar chlorophyll degradation in Romana lettuce. Collard greens stored in CH-TEO films incorporated with ZnO/PEG/NC/CaCl_2_ showed better retention of chlorophyll content under refrigerated storage conditions. This might be due to the lower oxygen transmission rate of biodegradable films incorporated with NC, ZnO, PEG, and CaCl_2_ which can prevent chlorophyll degradation^46,53,54^. Owolabi et al*.*^48^ reported that essential oil action on the surface of plant foods result in lower ethylene generation and respiration rates that minimize chlorophyllase activity. Thus the current findings revealed that CH-TEO films incorporated with ZnO/PEG/NC/CaCl_2_ delayed the reductions in chlorophyll levels from the initial values by retarding chlorophyll degradation and are a possible candidate for application to fresh-cut green leafy vegetables.

#### Total soluble solids and titratable acidity

**Figure 3:**
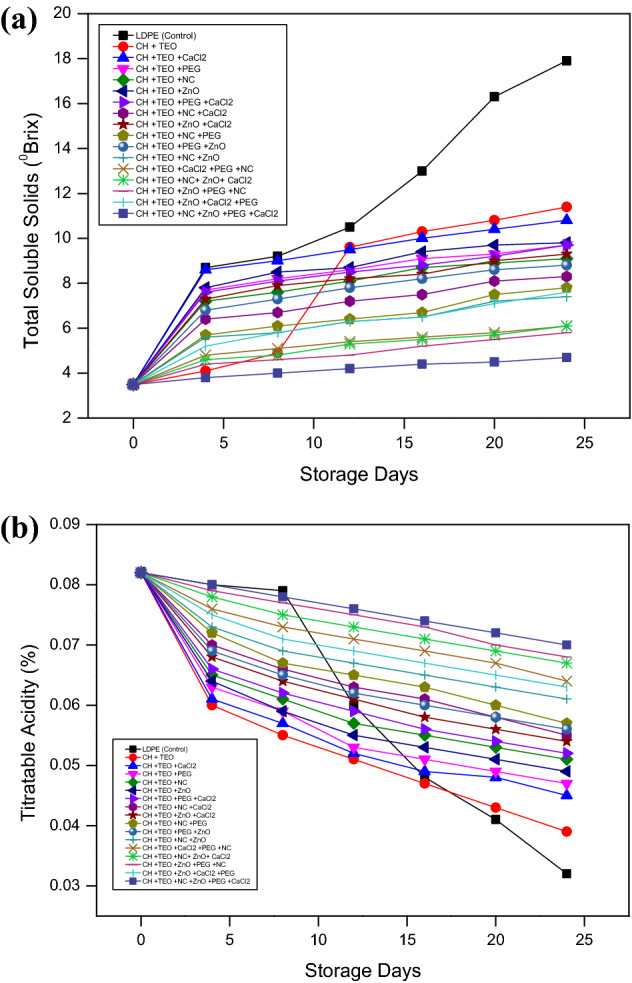
(**a**) Effect of different chitosan-based films and storage days on total soluble solids (°Brix) of collard greens. (**b)** Effect of different chitosan-based films and storage days on titratable acidity (%) of collard greens.

Total soluble solids (TSS) and titratable acidity (TA) of collard greens packaged under different biodegradable films including control were measured periodically at four days intervals up to 24 days (Fig. 3a,b). The results revealed that all collard green treatments including control exhibited an enhancement in TSS and reduction in TA with the advancement of postharvest storage up to 24 days. At the end of the storage period, collard greens packaged in CH-TEO + ZnO/PEG/NC/CaCl_2_ film (T_16_) showed lowest values of TSS and the highest TA contents among all treatments. TSS results from the breakdown of carbohydrates during fruit metabolism, and their content accelerates with increasing respiration rate^48^. The lower TSS of T_16_ treatment might be due to the slower hydrolysis of carbohydrates to sugars thus indicating slower respiration rate and metabolic activities because of the enhanced gas barrier (oxygen and carbon dioxide) properties of the film^55,56^. Thus, CH-TEO + ZnO/PEG/NC/CaCl_2_ film (T_16_), is better for retaining the TSS of collard greens than LDPE film (T_0_). Additionally, diffusion of volatile essential oil components to the fruit surface can inhibit TSS from increasing suddenly during storage^31^. The findings of this study are similar to those of Srinivasa et al.^57^ for tomato and bell pepper. Furthermore, TA content retention in collard greens packaged in CH-TEO films incorporated with ZnO/PEG/NC/CaCl_2_ may also be due to controlled CO_2_/O_2_ permeability^58^. Moreover, essential oils reduce the consumption of TA by decreasing the metabolic respiration rate of fruits during the storage period^48^. These results suggest that the preservation effect of ZnO/PEG/NC/CaCl_2_ film was better than the pure CH-TEO and LDPE films. Similar results were reported by Guo et al.^1^.

#### Color evaluation

**Figure 4:**
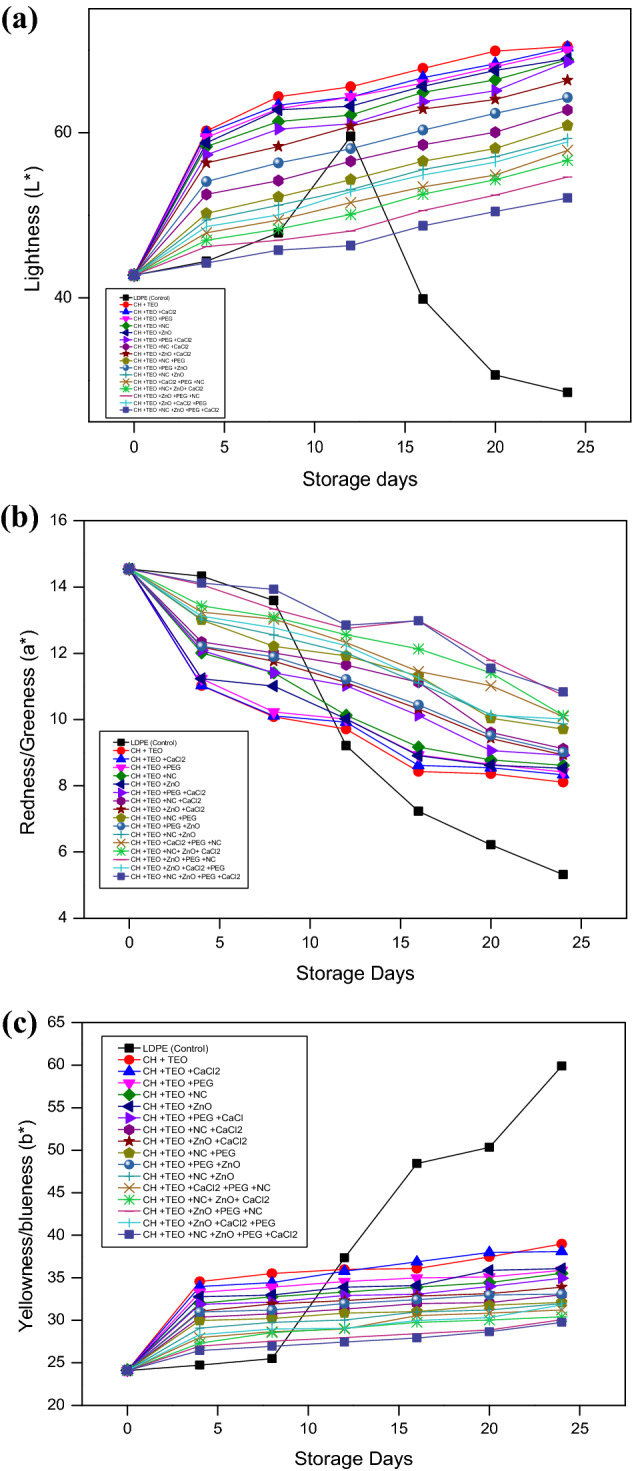
(**a**) Effect of different chitosan-based films and storage days on lightness value (L*) of collard greens. (**b)** Effect of different chitosan-based films and storage days on color (a* value) of collard greens. (**c)** Effect of different chitosan-based films and storage days on color (b* value) of collard greens.

Changes in leaf color is a significant qualitative factor that influences aesthetic quality of green leafy vegetables. However, color variations may occur in leafy vegetables mainly because of chlorophyll breakdown and browning triggered by processing or storage conditions. The lightness values (L*) of all the treatments (T_1_ to T_16_) increased throughout the storage period while, the slowest increase was observed in T_16_ treatment, indicating a slow depletion of the green color of the collard greens. However, control treatment (T_0_), the collard greens stored in LDPE film exhibited the greatest variation (p < 0.05) over the storage period which may be due to the appearance of mold growth after 8^th^ day of storage period. The L* values for collard greens packaged in biodegradable films showed that the CH-TEO-based films containing additives ZnO/PEG/NC/CaCl_2_ were more effective in keeping their initial green color. It agrees with a* and b* readings, indicating that the collard greens stored in CH-TEO + ZnO/PEG/NC/CaCl_2_ film were the greenish (lowest a* values) and the bluish (lowest b* values) at the end of the storage days (Fig. 4a–c). The changes in color parameters and chlorophyll content during storage can shorten shelf-life and are a critical problem for marketing fresh leafy vegetables. Hence, color variations and overall quality should be considered essential factors for the assessment of quality of fresh vegetables^59^. Overall, collard greens packed in CH-TEO-based films containing additives ZnO/PEG/NC/CaCl_2_ had the lowest L* and b* values. This result depicts that CH-TEO-based film containing additives ZnO/PEG/NC/CaCl_2_ has retarded chlorophyll degradation to carotene (pale yellow) and hydroxylated carotenoids (yellow). The results of this investigation showed that CH-TEO-based films containing additives ZnO/PEG/NC/CaCl_2_ preserved the color of collard greens during storage by inhibiting postharvest senescence Miceli et al.^60^.

#### Microbiological assessment

**Figure 5:**
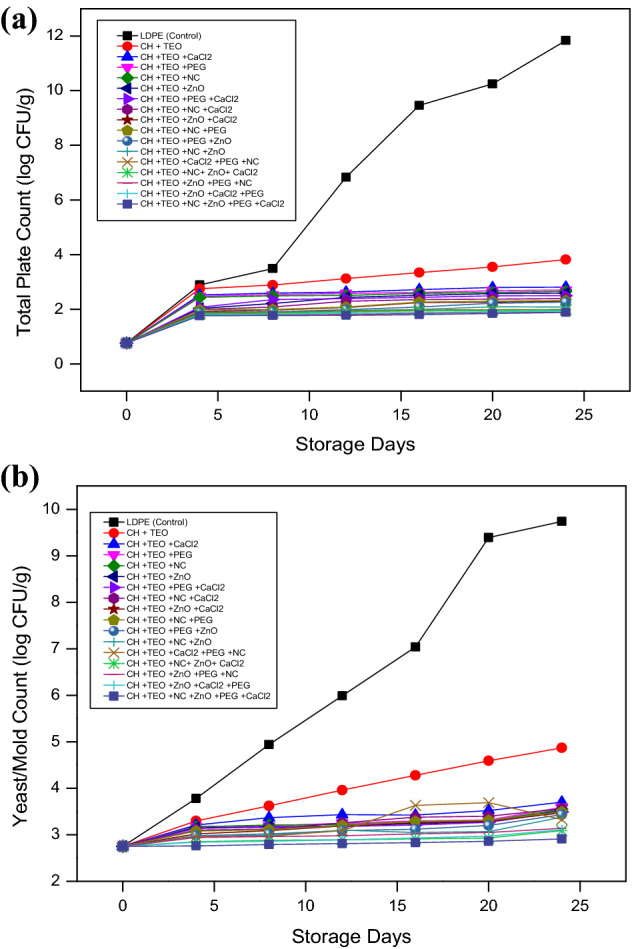
(**a**) Effect of different chitosan-based films and storage days on total plate count (log cfu/g) of collard greens. (**b)** Effect of different chitosan-based films and storage days on yeast and mold count (log cfu/g) of collard greens.

Total plate count and yeast/mold count of collard greens packaged in different biodegradable films are shown in Fig. 5a,b. The collard greens stored in control film (T_0_) did not exhibit any inhibitory effect. Among the biodegradable films, T_1_ showed the least antimicrobial activity while T1_6_ exhibited the highest antimicrobial activity. CH has an inherent antimicrobial capability^61^. Several studies have shown that the biological activity of CH depends significantly on its molecular weight (MW). The low-MW CH not only has extracellular antimicrobial activity but also intracellular antimicrobial activity, thereby affecting RNA, protein synthesis, and mitochondrial function^62–64^. CH-based films incorporated with TEO or ZnO/PEG/NC/CaCl_2_ exhibited significant inhibition in total plate count and it increased significantly (p < 0.05) by adding ZnO/PEG/NC/CaCl_2_ contents. This achievement might be linked to the synergistic effect of TEO and ZnO/PEG/NC/CaCl_2_ as reported by previous literature^65^. The antimicrobial activity of T_16_ CH film against bacteria, yeast and molds were highest because of the binding of positively charged amino groups of CH to the primarily anionic components of the bacteria surface^66^. Furthermore, the antimicrobial activity of CH films containing additives viz., ZnO/PEG/NC/CaCl_2_ might be attributed to a number of mechanisms, including the interaction of nanoparticles with bacterial surfaces and cell membrane contact, ion penetration into the cell membrane, and reaction with sulfhydryl groups inside the cell membrane, disruption of cell division and thus microorganism death^67^. There was a further enhancement in the antimicrobial activity by the addition of TEO mainly by its phenolic compounds, such as thymol and carvacrol^68^. In general, bacteria have a higher sensitivity to essential oils than molds and yeasts^69^. These phenolic chemicals may cause bacterial outer membrane breakdown, resulting in the release of lipopolysaccharides and enhanced cytoplasmic membrane permeability^70^. The findings of the present study are in agreement with Guo et al.^1^ for cherry tomatoes. Brandelero et al.^49^ also observed that the lettuce stored in the biodegradable films with lemongrass oil as antimicrobial agent had shelf life of 4 days whereas the lettuce stored in the PVC film had shelf life of only 2 days only.

#### Sensory evaluation

**Figure 6:**
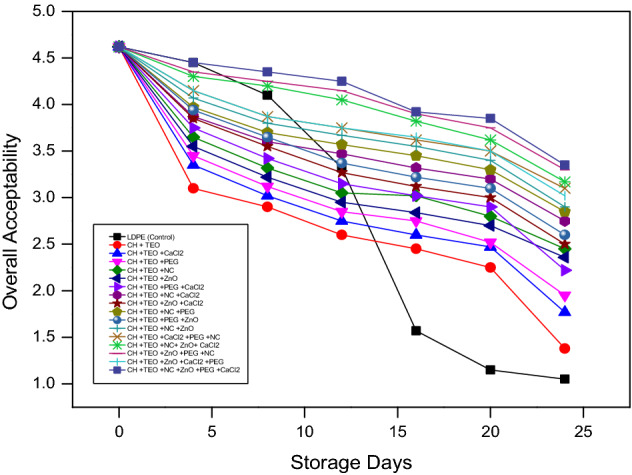
Effect of different chitosan-based films and storage days on overall acceptability of collard greens.

Sensory evaluation of collard greens packaged in different combinations of biodegradable films was subjectively conducted by a trained panel of 10 individuals who rated overall acceptability (Fig. 6). The results showed that, regardless of the treatments, overall acceptability scores decreased significantly from day 1 to day 24 (p < 0.05). Although, the highest decrease in overall acceptability scores was observed in collard greens stored in control films as compared to the collard greens packaged in various active biodegradable CH based films. After 24 days of the storage period, the highest mean score for overall acceptability was observed in collard greens packaged in CH-TEO + ZnO/PEG/NC/CaCl_2_ film due to the retention of green color and leaf stiffness after 8 days of storage. The CH-TEO-based film containing ZnO, PEG, NC, and CaCl_2_ establishes a barrier in the route of moisture and gases (oxygen and carbon dioxide) thus, minimizes excessive degradation of biological components (chlorophyll) and alteration in physical properties such as TSS and TA. ZnO is indeed an antioxidant with a lot of potential for preserving a treated produce's postharvest quality^71^. As a result, the CH-TEO films incorporated with ZnO/PEG/NC/CaCl_2_ preserved their color longer compared to the other films including control throughout the storage time. The samples stored in the biodegradable films retained green color up to 14^th^ day of storage presenting acceptable color whereas in LDPE, the sample retained its color up to 8^th^ day of storage. Hence, color variations and overall quality should be considered essential factors for the quality evaluation of fresh leafy green vegetables.

## Conclusions

The results concluded that CH-TEO-based films incorporated with ZnO/CaCl_2_/NC/PEG are effective in the conservation of postharvest quality of collard greens during refrigerated storage. The prepared composite films (CH-TEO + ZnO/PEG/NC/CaCl_2_) were having low water vapor transmission rate, high tensile strength, and were water soluble and biodegradable in nature. In addition, collard greens packaged in CH-TEO incorporated with ZnO/PEG/NC/CaCl_2_ films showed less physiological weight loss, retained total soluble solids, and titratable acidity as well as showed lesser a* values, suppressed microbial growth, and preserving the overall acceptability of collard greens at least 24 days gain in the commercial life in comparison to LDPE films. In conclusion, the incorporation of ZnO/PEG/NC/CaCl_2_ in CH-TEO-based films is a useful approach in maintaining the quality of harvested collard greens for a longer period under refrigerated conditions.

## Supplementary Information

Supplementary Information.

## Data Availability

The datasets generated and/or analyzed during the current study are not publicly available due to privacy concerns but are available from the corresponding author upon reasonable request.
