# Supplementary material for: Development of chitosan-based biodegradable films enriched with thyme essential oil and additives for potential applications in packaging of fresh collard greens
Source: Sci Rep. 2022 Oct 8;12:16923. doi: 10.1038/s41598-022-20751-1 (PMC9547897; doi:10.1038/s41598-022-20751-1)
Supplement: Supplementary file 1 — Supplementary Information. [file 41598_2022_20751_MOESM1_ESM.docx]

| 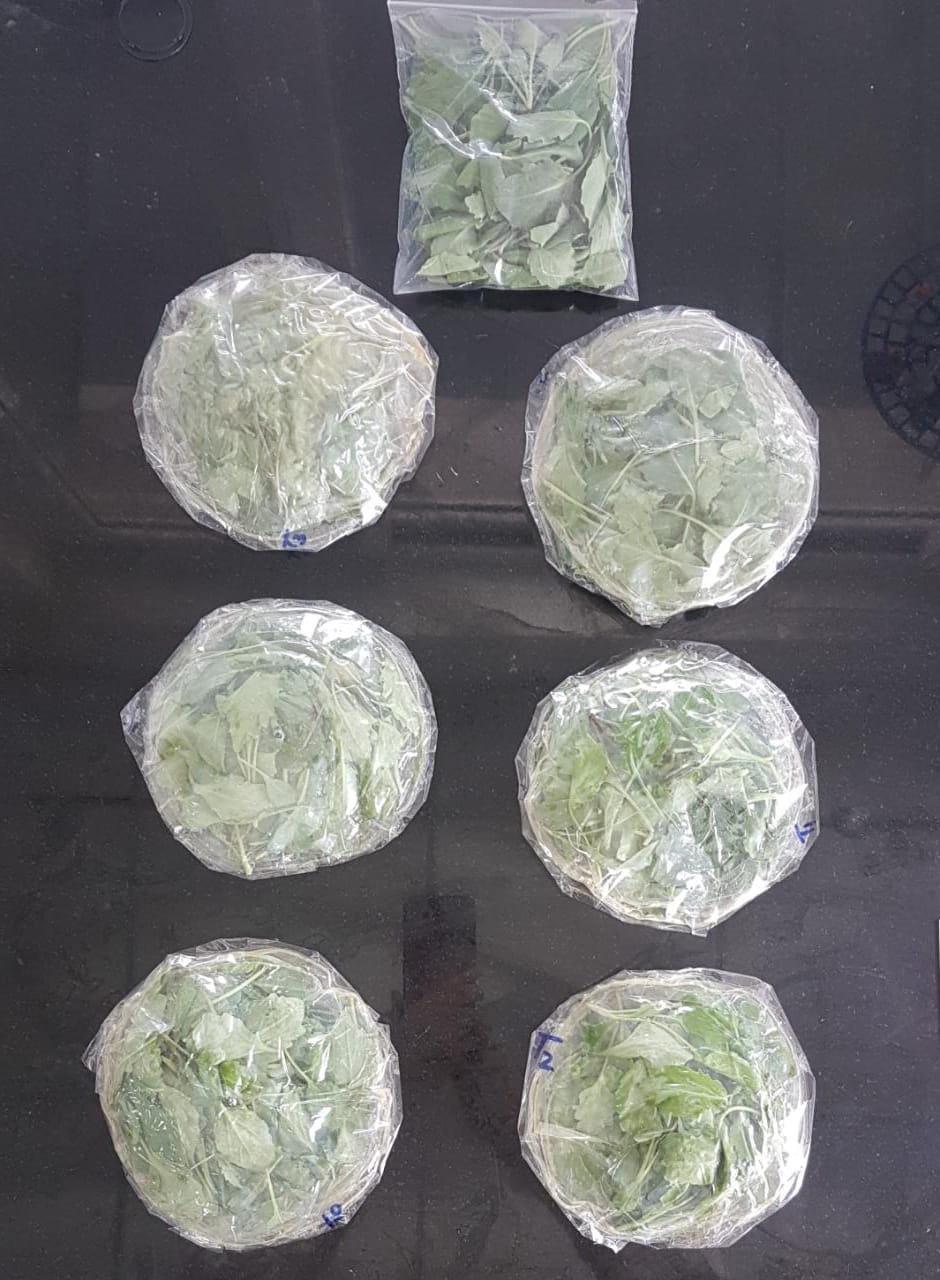 | 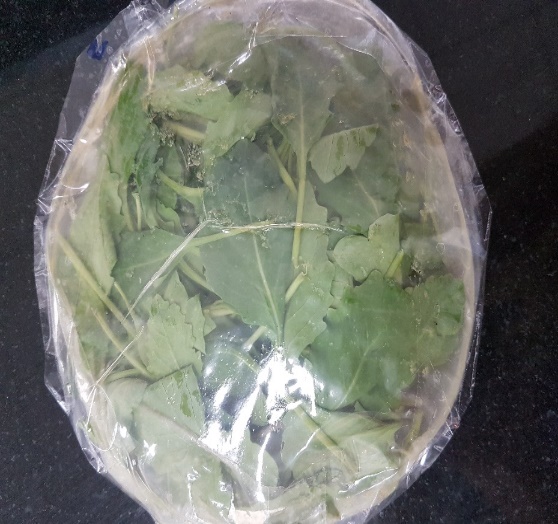 | 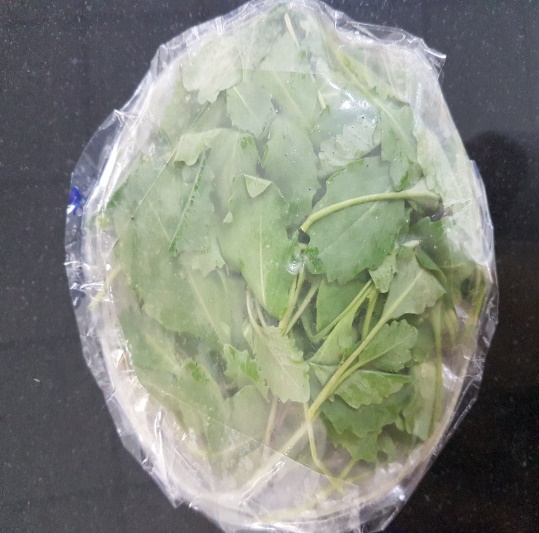 |
| --- | --- | --- |
| (T_0_) LDPE Film | (T_1_) Chitosan (CH) +Thyme oil (TO) | (T_2_) CH +TO +Calcium chloride (CaCl_2_) |
| 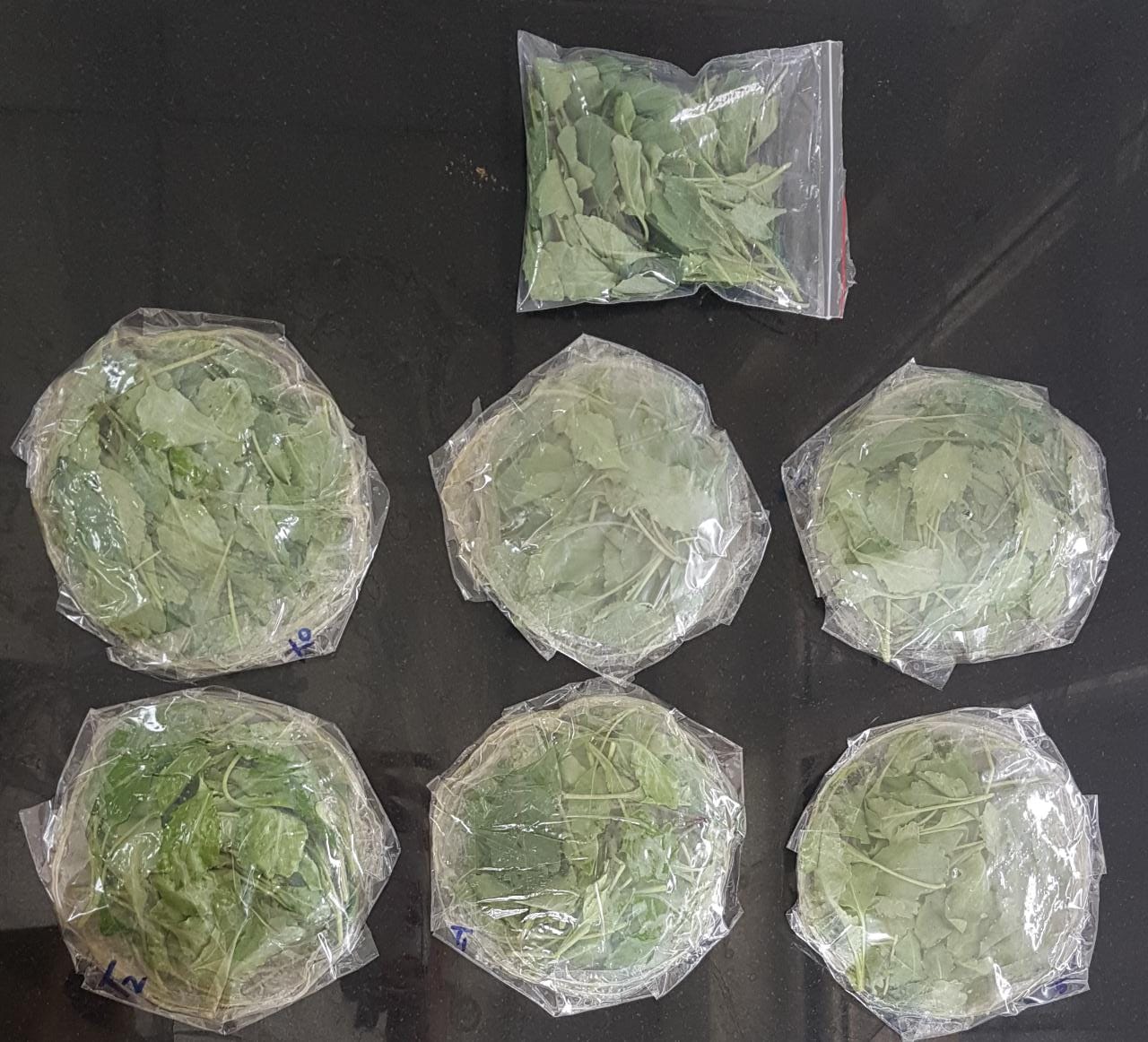 | 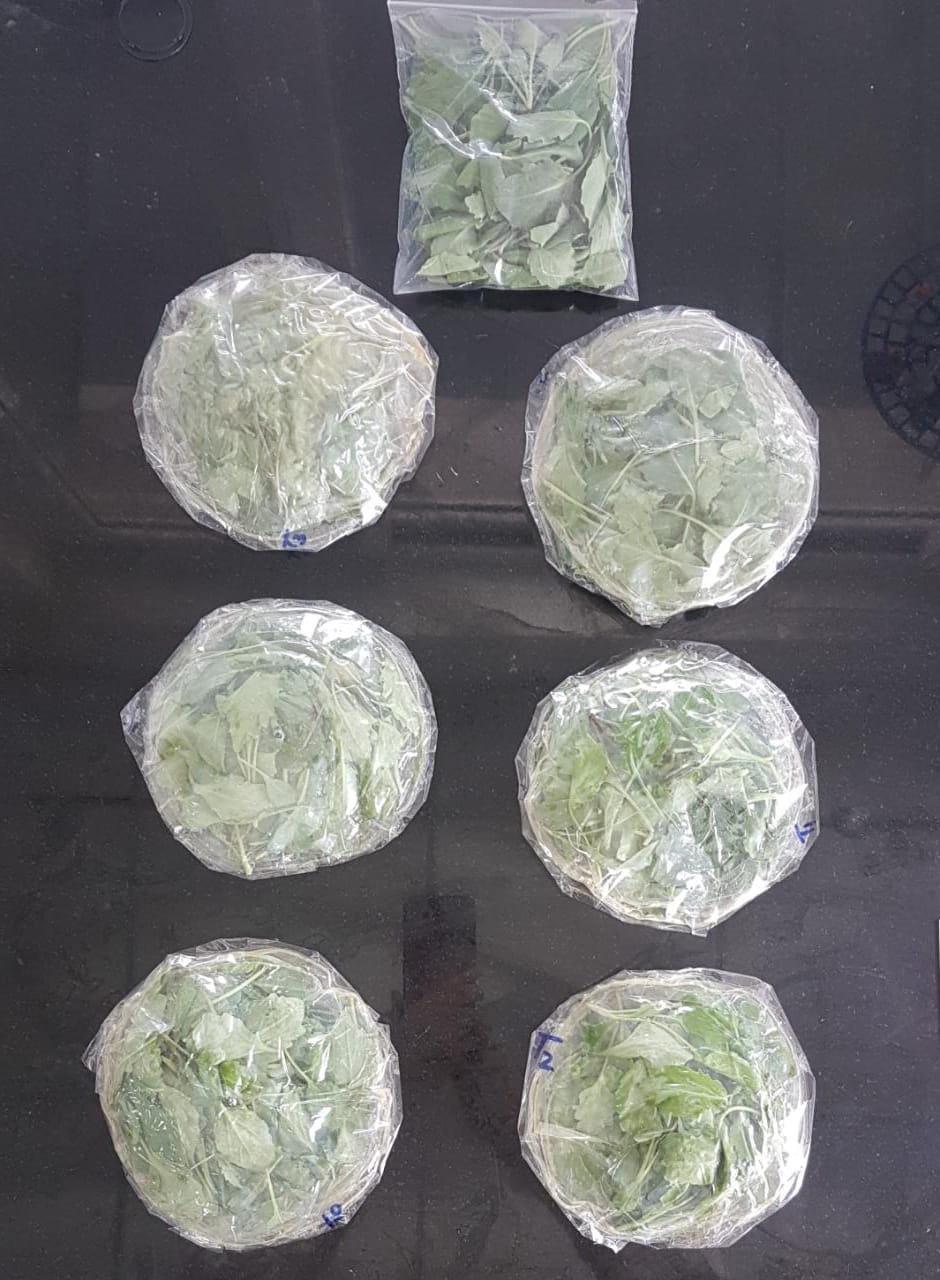 | 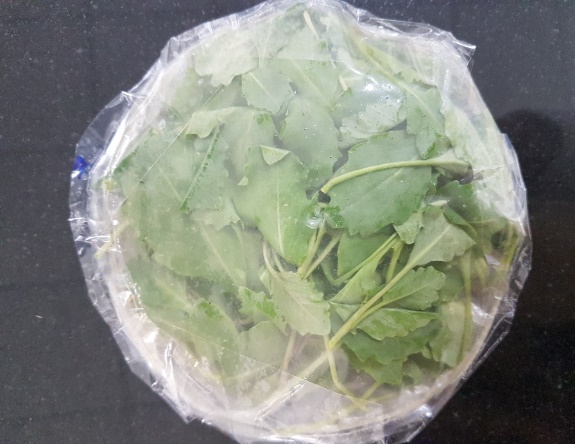 |
| (T_3_) CH +TO +Polyethylene glycol (PEG) | (T_4_) CH +TO +Nano-clay (NC) | (T_5_) CH +TO +Zinc-oxide (ZnO) |
| **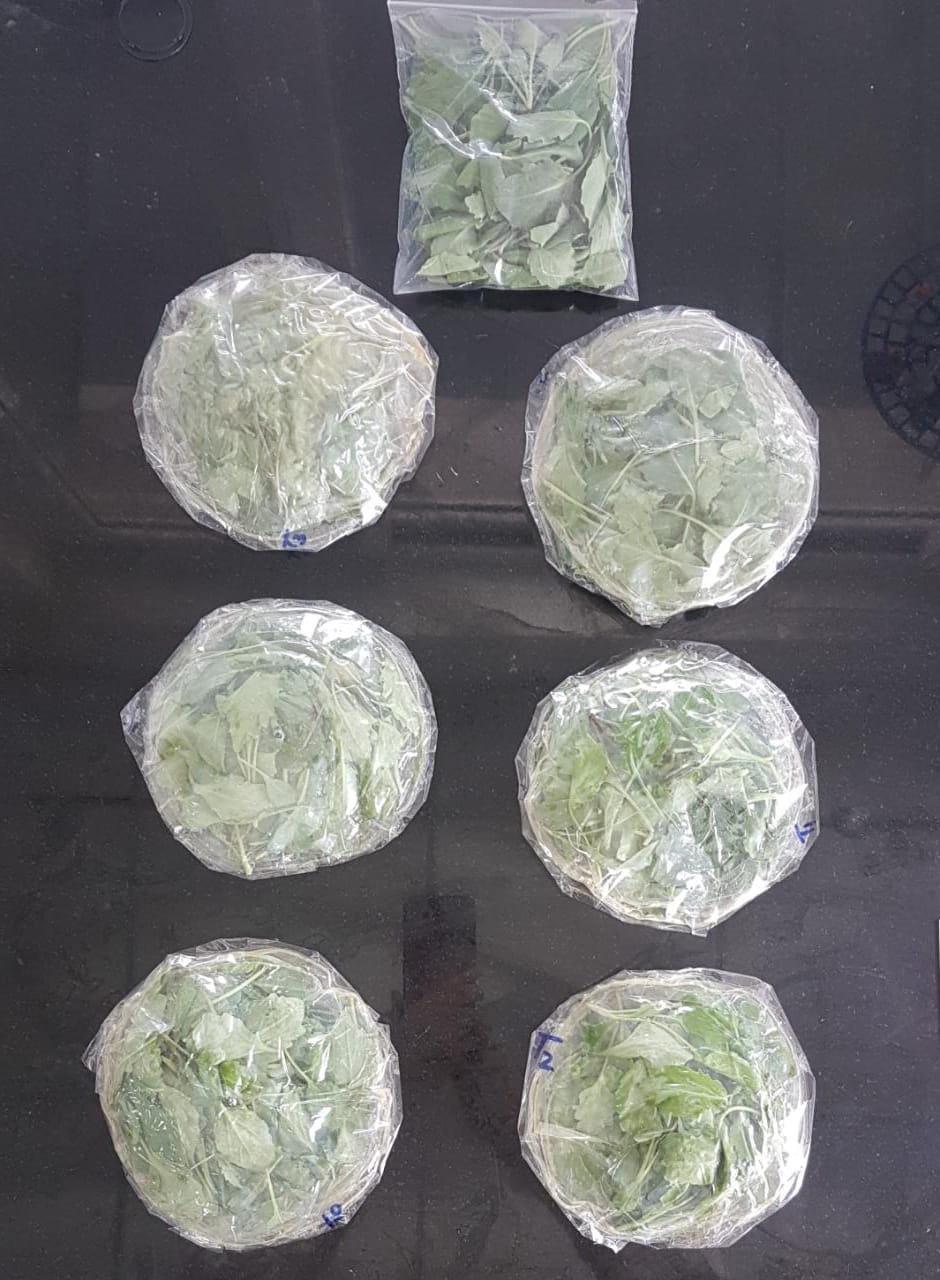** | 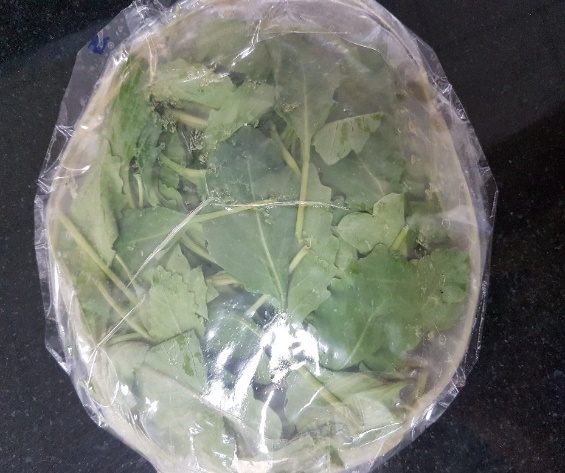 | 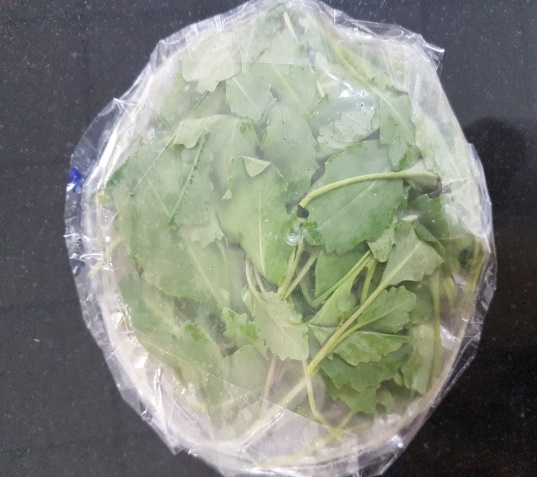 |
| (T_6_) CH +TO +PEG +CaCl_2_ | (T_7_) CH +TO +NC +CaCl_2_ | (T_8_) CH +TO +ZnO +CaCl_2_ |
| **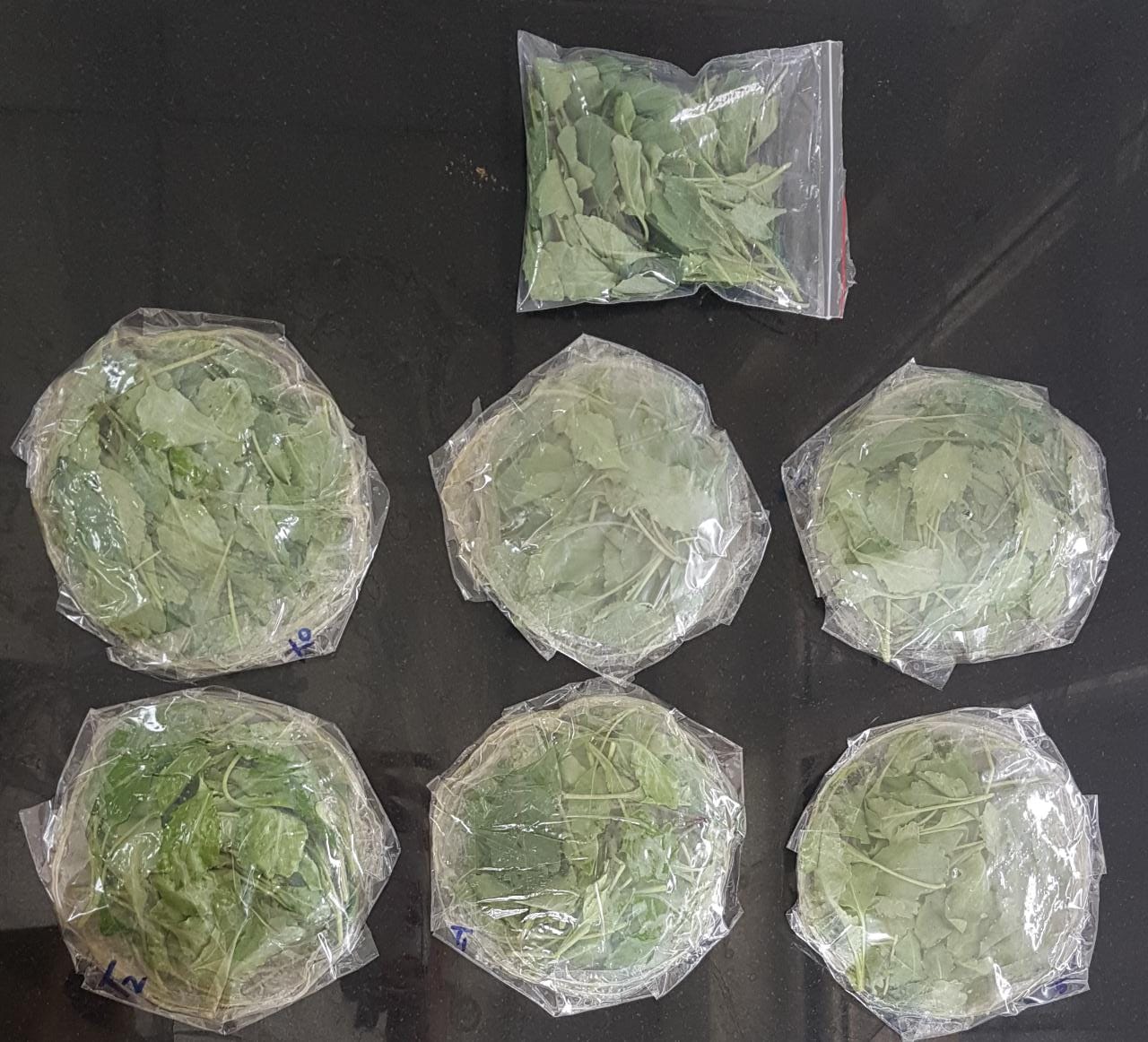** | 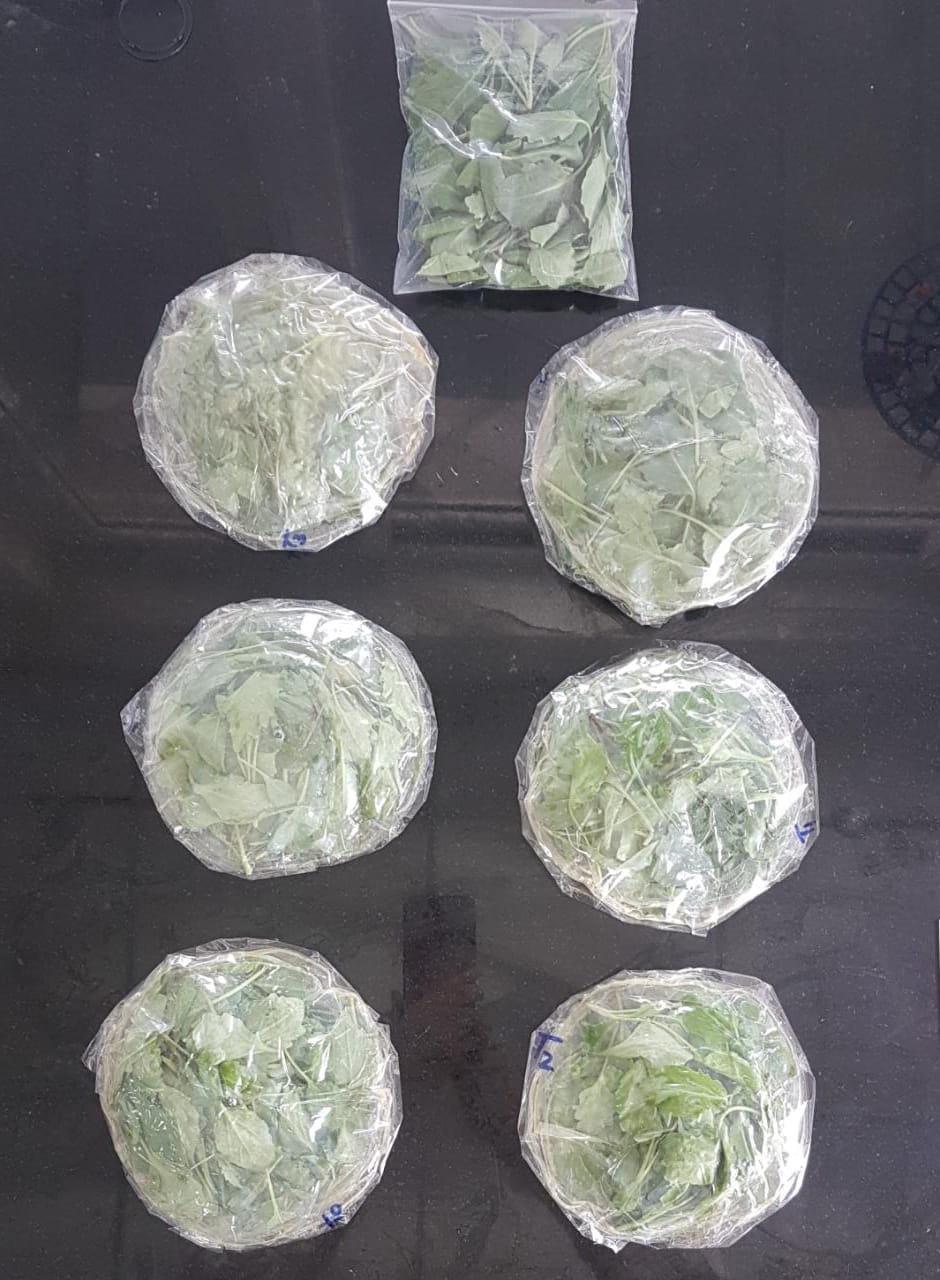 | 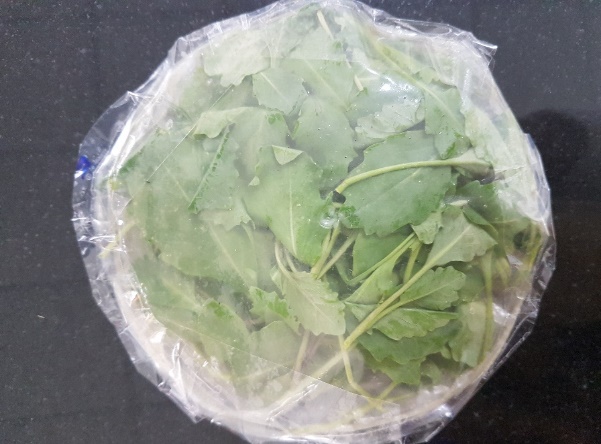 |
| (T_9_) CH +TO +NC +PEG | (T_10_) CH +TO +PEG +ZnO | (T_11_) CH +TO +NC +ZnO |
| 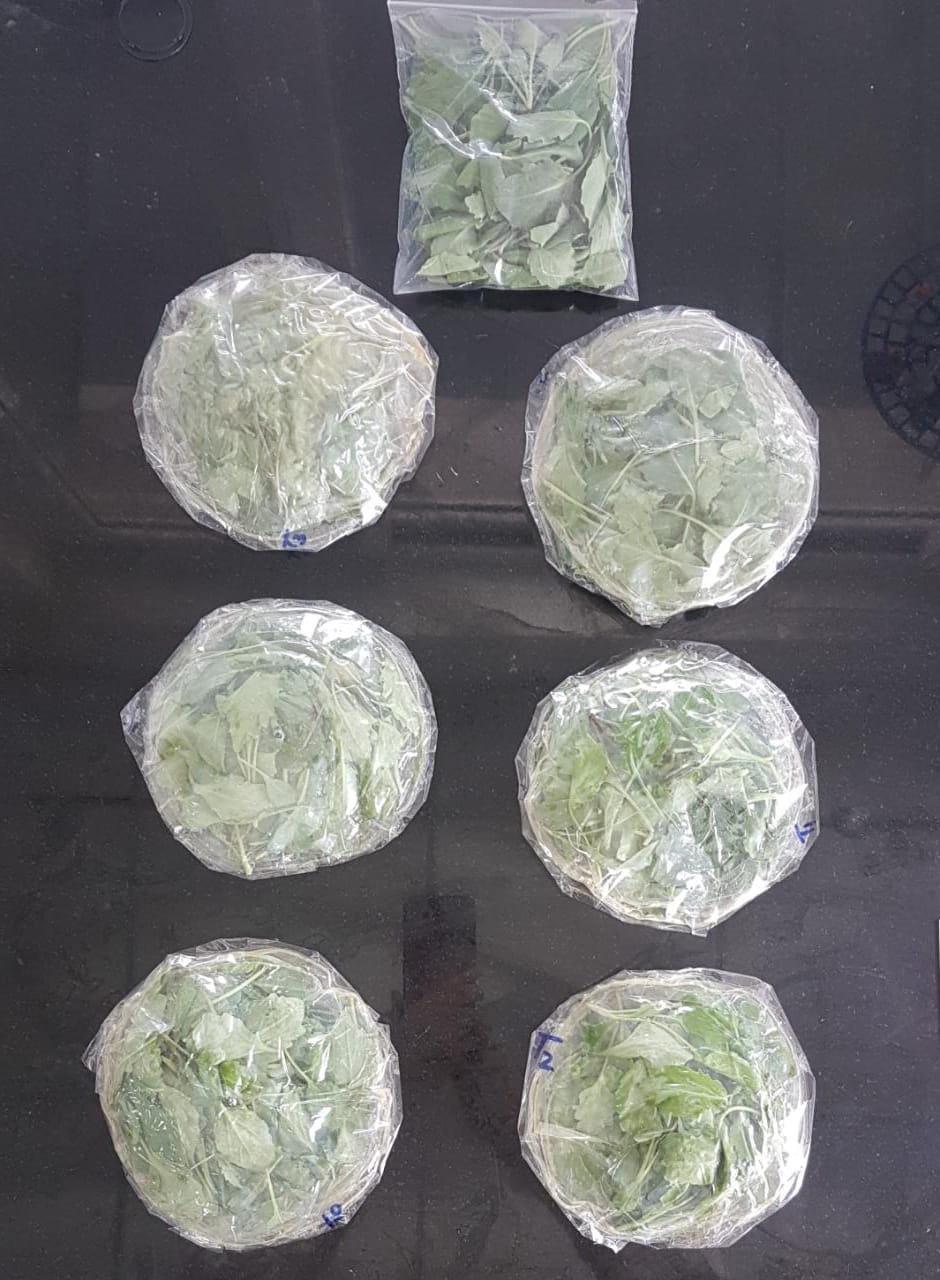 | 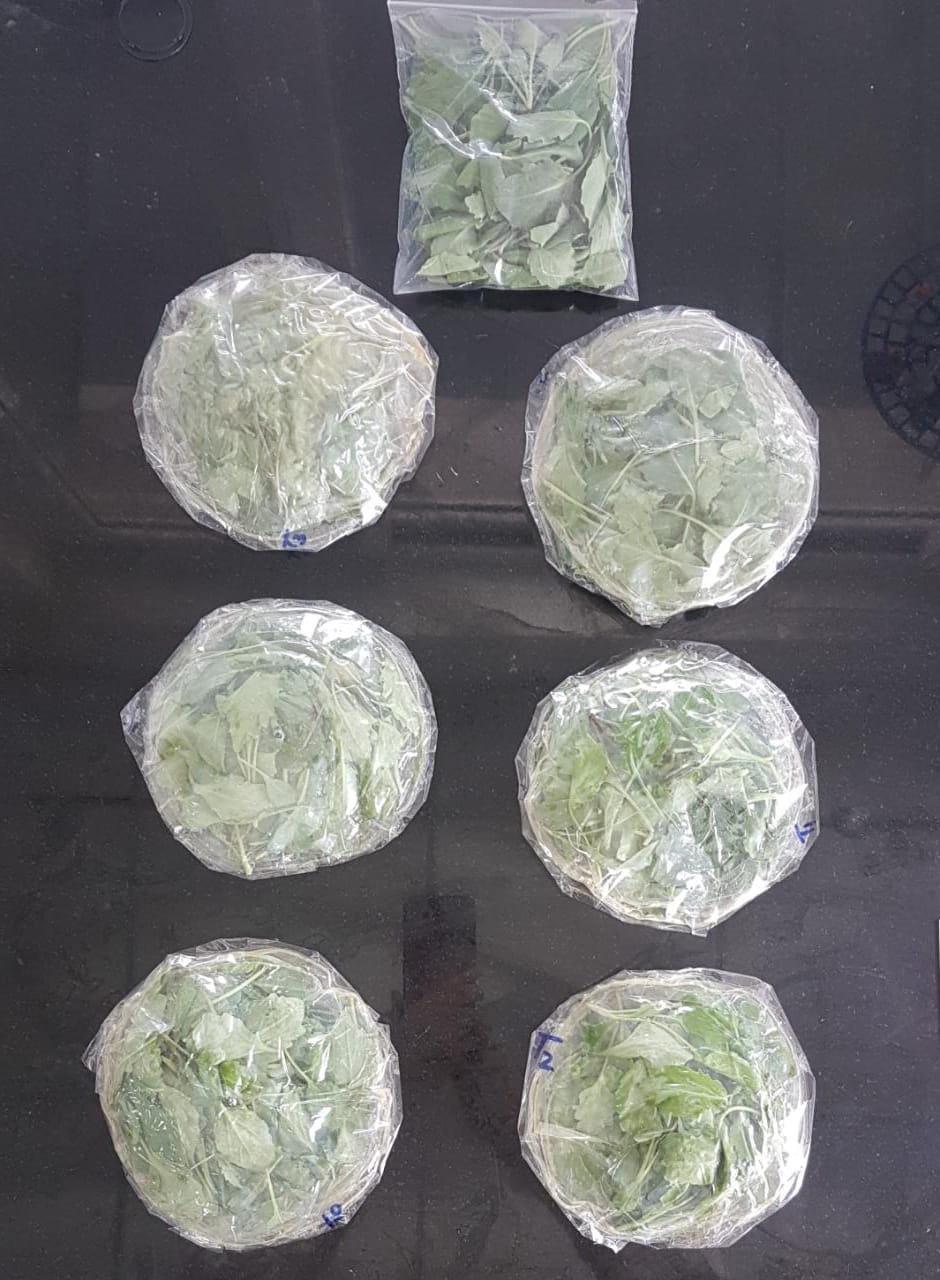 | 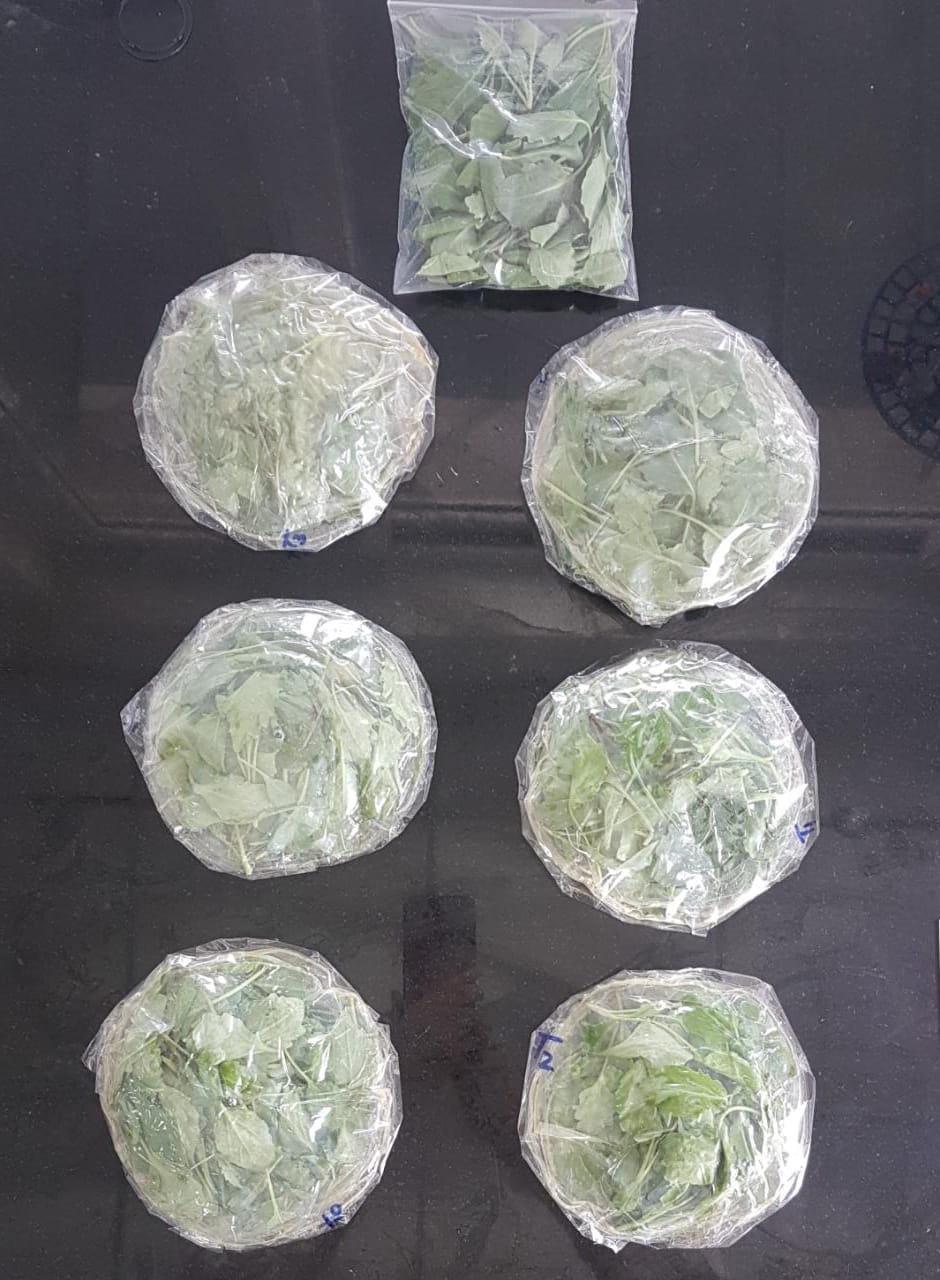 |
| (T_12_) CH +TO +CaCl_2_ +PEG +NC | (T_13_) CH +TO +NC+ ZnO+ CaCl_2_ | (T_14_) CH +TO +ZnO +PEG +NC |
| 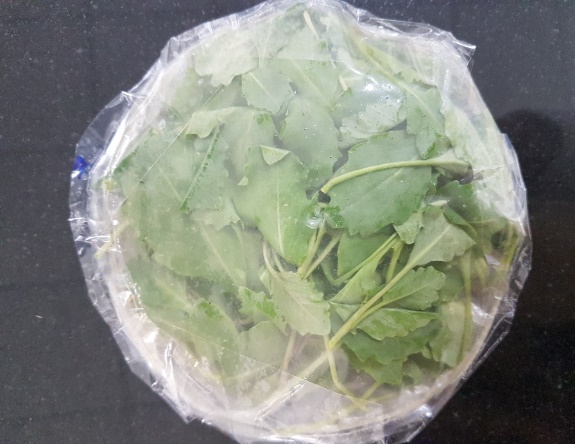 | 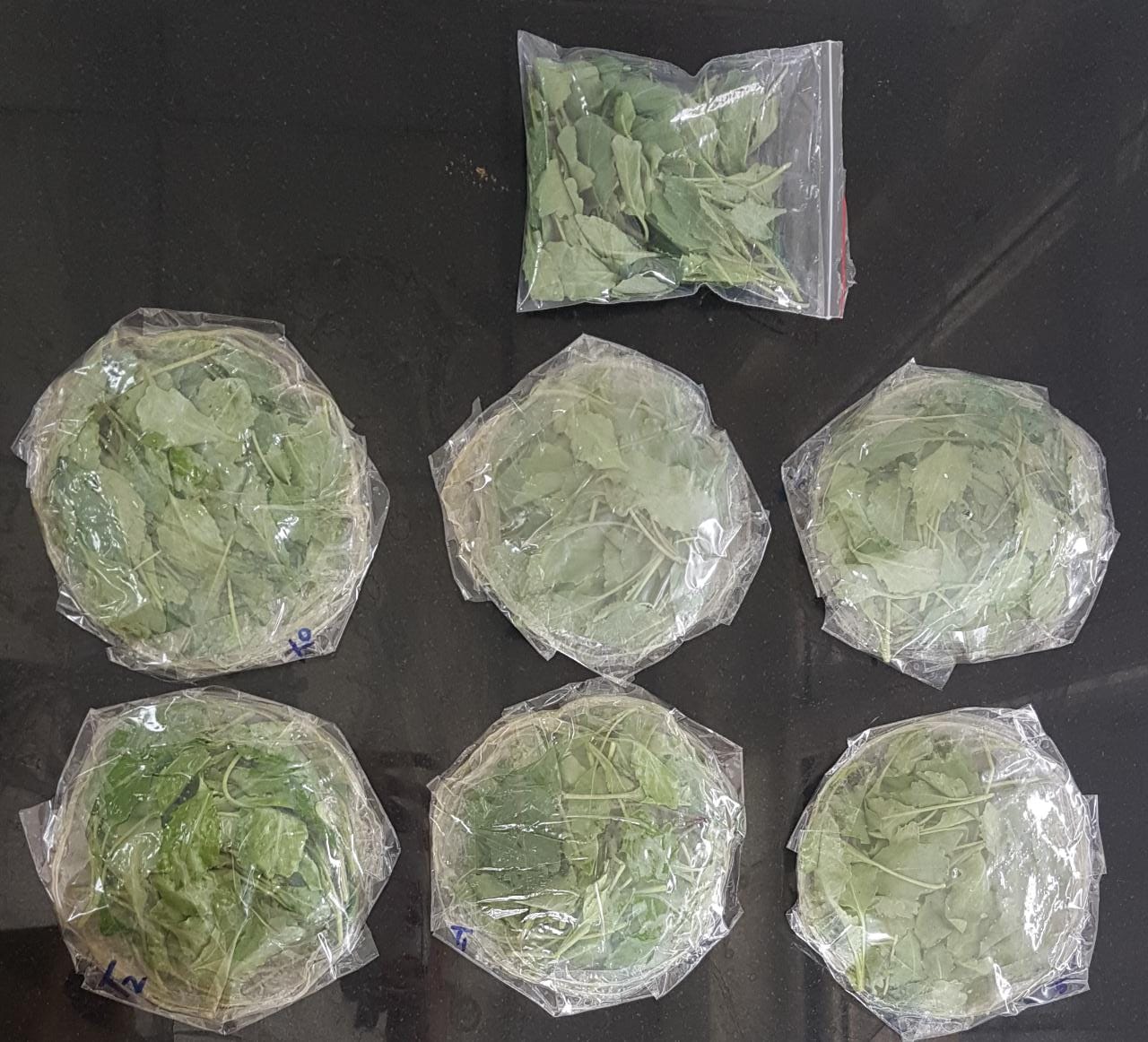 |  |
| (T_15_) CH +TO +ZnO +PEG +NC | (T_16_) CH +TO +NC +ZnO +PEG +CaCl_2_ |  |

**Plate S:** Storage of collard greens in different chitosan-based packaging films
